# Profiling of Concanavalin A-Binding Glycoproteins in Human Hepatic Stellate Cells Activated with Transforming Growth Factor-β1

**DOI:** 10.3390/molecules191219845

**Published:** 2014-11-28

**Authors:** Yannan Qin, Yaogang Zhong, Ganglong Yang, Tianran Ma, Liyuan Jia, Chen Huang, Zheng Li

**Affiliations:** 1 Department of Genetics and Molecular Biology, Xi’an Jiaotong University College of Medicine, Xi’an 710061, Shaanxi, China; E-Mail: yannan159@mail.xjtu.edu.cn; 2 Laboratory for Functional Glycomics, College of Life Sciences, Northwest University, Xi’an 710069, Shaanxi, China; E-Mails: zhongyglyco@163.com (Y.Z.); glyanglife@hotmail.com (G.Y.); rooroo@163.com (T.M.); csshxzza@163.com (L.J.)

**Keywords:** ConA-binding glycoprotein, hepatic stellate cells, transforming growth factor-β1, mass spectrometry, peptides, KEGG pathway

## Abstract

Glycoproteins play important roles in maintaining normal cell functions depending on their glycosylations. Our previous study indicated that the abundance of glycoproteins recognized by concanavalin A (ConA) was increased in human hepatic stellate cells (HSCs) following activation by transforming growth factor-β1 (TGF-β1); however, little is known about the ConA-binding glycoproteins (CBGs) of HSCs. In this study, we employed a targeted glycoproteomics approach using lectin-magnetic particle conjugate-based liquid chromatography-tandem mass spectrometry to compare CBG profiles between LX-2 HSCs with and without activation by TGF-β1, with the aim of discovering novel CBGs and determining their possible roles in activated HSCs. A total of 54 and 77 proteins were identified in the quiescent and activated LX-2 cells, respectively. Of the proteins identified, 14.3% were glycoproteins and 73.3% were novel potential glycoproteins. Molecules involved in protein processing in the endoplasmic reticulum (e.g., calreticulin) and calcium signaling (e.g., 1-phosphatidylinositol-4,5-bisphosphate phosphodiesterase β-2 [PLCB2]) were specifically identified in activated LX-2 cells. Additionally, PLCB2 expression was upregulated in the cytoplasm of the activated LX-2 cells, as well as in the hepatocytes and sinusoidal cells of liver cirrhosis tissues. In conclusion, the results of this study may aid future investigations to find new molecular mechanisms involved in HSC activation and antifibrotic therapeutic targets.

## 1. Introduction

Liver fibrosis is a major complication of various chronic hepatic diseases, resulting from increased production and decreased degradation of the extracellular matrix. Activation and phenotypic transformation of hepatic stellate cells (HSCs) play a central role in the development and resolution of liver fibrosis and cirrhosis [1,2], and in the promotion of hepatocellular carcinoma [3]. Transforming growth factor-β1 (TGF-β1), as the key fibrogenic cytokine [4,5], activates HSCs via the TGFβ1/activin receptor-like kinase 1 (ALK1)/Smad1 pathway and stimulates collagen transcription in HSCs [6].

Glycosylation is the major posttranslational modification to the secretory and the membrane-anchored proteins in eukaryotic cells [7], which plays important roles in cell-cell adhesion, bacterial infection, viral attachment, ligand-receptor binding, and other key cellular processes [8]. In recent years, a growing number of studies have reported that protein glycosylations are frequently altered during physiological and pathological changes [9,10,11]. Many receptor–ligand interactions lead to alteration of protein-bound glycans, and numerous receptors are regulated by glycosylation to a lesser or greater degree; for example, Notch receptors are the recipient substrates of specific glycosyltransferases [12,13], which regulate the activation of Notch by its ligands and thus affect various processes during development. In HSCs, interleukin-1 and tumor necrosis factor-α have lectin-like activities [14] and recognize disialylated diantennary N-glycans bearing two Neu5Aca2,3 residues [15] and heparin [16,17], respectively. Our previous study also indicated that concanavalin A (ConA) and six other lectins showed stronger binding to the activated HSCs than to the quiescent HSCs [18].

Lectins are carbohydrate-binding proteins that discriminate terminal glycans based on subtle differences in their structures, which can be exploited in the laboratory to detect or isolate specific carbohydrate structures of glycoconjugates, including glycoproteins and glycolipids as well as glycosaminoglycans [19,20]. Along with the rapid improvement of cutting-edge separation technologies coupled with mass spectrometry in the fields of glycoproteomics and glycomics, glycoproteins can be enriched for analysis by hydrazide chemistry [21,22], lectin affinity [23,24], immunoaffinity [25,26], boric acid chemistry [27,28], size exclusion chromatography [29,30], hydrophilic interaction [31,32], and other methods. Lectin affinity chromatography is a unique method that can isolate glycoproteins/glycopeptides containing similar terminal glycan structures from complex samples.

In this study, we employed a targeted glycoproteomics approach using lectin-magnetic particle conjugate-based liquid chromatography-tandem mass spectrometry (LC-MS/MS) to compare the profiles of ConA-binding glycoproteins (CBGs) between human HSCs (LX-2 cell line) with and without activation by TGF-β1. The aim of this study was to determine whether any novel CBGs may be differentially expressed in the activated, compared with quiescent LX-2 cells, and consider the potential roles that these CBGs may play in the pathophysiology of liver fibrosis.

## 2. Results and Discussion

### 2.1. Isolation of Con A-Binding Glycoproteins (CBGs) from LX-2 Cells

**Figure 1:**
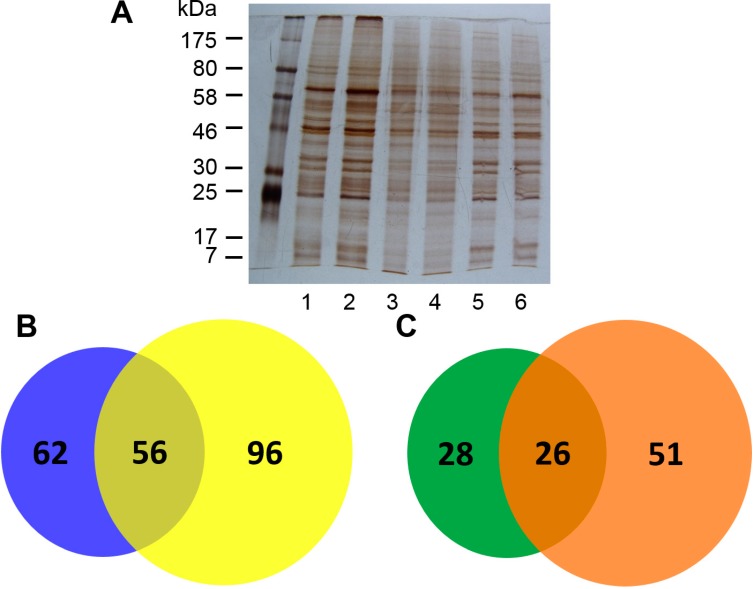
SDS-PAGE analysis and MS identification of the isolated CBGs. (**A**) Proteins were resolved on 10% SDS gels and stained with silver staining. Lanes 1 and 2 showed total proteins, lanes 3 and 4 showed the unbound proteins, and lanes 5 and 6 showed the enriched CBGs from the quiescent and the activated LX-2 cells, respectively; (**B**) The Venn diagrams present the numbers of peptides and glycoproteins identified by MS. Left, the quiescent LX-2 cells; right, the activated LX-2 cells.

ConA-magnetic particle conjugates (CMPCs) were used to isolate CBGs from total protein lysates of the activated and quiescent LX-2 cells. The total, unbound, and eluted proteins were resolved by SDS-PAGE and visualized by silver staining. The results showed that the bands of CBGs (Figure 1A, lanes 5 and 6) were complementary to those of the unbound proteins (lanes 3 and 4), and that the eluted CBGs were enriched to some degree from the total proteins (lanes 1 and 2) (Figure 1A).

### 2.2. Identification of CBGs Isolated by CMPCs by LC-MS/MS

CBGs isolated by CMPCs from total proteins of the activated and quiescent LX-2 cells were identified by Agilent 6530 accurate-mass Q-TOF LC-MS/MS. A total of 118 and 152 unique peptides were identified, respectively, from the quiescent and activated LX-2 cells, and these peptides represented 54 and 77 proteins, respectively (Figure 1B,C). Then, the percentage amounts by which emPAI-based protein abundance estimates deviated from the actual measured values were calculated to permit the analysis and comparison of protein expression between the activated and quiescent LX-2 cells. As a result, four proteins (calreticulin [CALR], histone H4, galectin [LGALS]1, and isoform 1 of nucleoside diphosphate kinase A) were upregulated (fold-change > 2), and two proteins (14-3-3 protein ζ/δ and protein disulfide-isomerase) were downregulated in the activated LX-2 cells (fold-change < 0.5). In addition, 28 proteins (including 14-3-3 protein ε, protease serine 4 isoform B, and 14-3-3 protein γ) were specifically identified in the quiescent LX-2 cells and 51 proteins (including isoform 2 of procollagen-lysine, 2-oxoglutarate 5-dioxygenase 2, POTE ankyrin domain family member F, and vimentin) were specifically identified in the activated LX-2 cells. In total, by mapping to the Uniprot database, 13 (12.4%) of the identified proteins were classified as unreviewed proteins, 15 proteins (14.3%) were classified as glycoproteins, and 77 proteins (73.3%) were classified as unknown proteins (Table 1). According to two online glycosylation site prediction servers (NetNGlyc 1.0 [33] and NetOGlyc 4.0 [34]), 62 of the 77 “unknown” proteins were predicted to have potential N-glycosylation sites, and 15 proteins were predicted to have potential O-glycosylation sites. There were still three proteins that were not identified as glycoproteins by the online servers (Table 1).

Lectins are defined as carbohydrate-binding proteins that are neither antibodies nor enzymes, which have a wide range of glycan-binding specificities, and are therefore suitable for the partial isolation and characterization of a glycome. ConA is a lectin originally extracted from the jack-bean *Canavalia ensiformis*, which binds specifically to internal and nonreducing terminal α-d-mannosyl (α-Man) and α-d-glucosyl groups (α-Glu) [35]. In this study, two possible consequences of the affinity attachment of CBGs to ConA were considered. First, total proteins were extracted under non-denaturing conditions; therefore, some non-target proteins that interacted with CBGs might have been pulled down together. Second, ConA is known to be a glycoprotein containing N-glycans linked to the 152th amino acid (Asn) for each sub-unit [36], and therefore, it was inevitable that cellular glycan-binding proteins with an affinity for the glycans of ConA might also have been collected by CMPCs. These two factors, together with the addition of the possible unusual O-glycosylations (e.g., O-mannosylation and O-GlcNAc) of the CBGs may explain the identification of some “non-glycoproteins” by MS (Table 1). To solve those two problems as effectively as possible, denaturing rinses were conducted several times with 0.1% (v/v) Tween^®^ 20, and specific competitive elution was performed with 0.5 M methyl-α-d-mannose and 0.2 M methyl-α-d-glucose.

**Table 1:** Detailed information about the CBGs (ConA-binding glycoproteins) isolated by CMPCs (ConA-magnetic particle conjugates) from activated and quiescent LX-2 cells.

No.	Accession	Description	Gene Name	Search Score	Coverage %	A/Q Ratio ^a^	Q or A ^b^	Known ^c^
1	IPI00003865	Isoform 1 of Heat shock cognate 71 kDa protein	HSPA8	375	26.8	—	Q, A	P^N^
2	IPI00022434	Putative uncharacterized protein ALB	ALB	260	6.5	—	Q, A	Y^N^
3	IPI00021263	14-3-3 protein zeta/delta	YWHAZ	164	15.1	0.16	Q, A	P^N^
4	IPI00010796	Protein disulfide-isomerase	P4HB	109	17.5	0.5	Q, A	P^N^
5	IPI00011134	Putative heat shock 70 kDa protein 7	HSPA7	96	6.5	—	Q, A	P^N^
6	IPI00220327	Keratin, type II cytoskeletal 1	KRT1	92	3.4	—	Q, A	P^N^
7	IPI00009865	Keratin, type I cytoskeletal 10	KRT10	75	14.6	—	Q, A	P^N^
8	IPI00217966	Isoform 1 of L-lactate dehydrogenase A chain	LDHA	68	4.5	—	Q, A	P^N^
9	IPI00021439	Actin, cytoplasmic 1	ACTB	65	17.9	—	Q, A	P^N^
10	IPI01969230	LOC644914; H3F3B; LOC440926; H3F3A Histone H3;xx	Null	60	8.9	—	Q, A	Null
11	IPI00418471	Vimentin	VIM	56	2.1	—	Q, A	Y°
12	IPI00020599	Calreticulin	CALR	53	10.8	2.44	Q, A	Y^N^
13	IPI00183968	tropomyosin alpha-3 chain isoform 1	TPM3	53	7.4	—	Q, A	P^N^
14	IPI00218820	Isoform 3 of Tropomyosin beta chain	TPM2	53	8.9	—	Q, A	P^N^
15	IPI00293665	Keratin, type II cytoskeletal 6B	KRT6B	49	4.4	—	Q, A	P^N^
16	IPI00453473	Histone H4	HIST1H4H	36	29.1	3.13	Q, A	P°
17	IPI00003269	Beta-actin-like protein 2	ACTBL2	32	6.6	—	Q, A	P^N^
18	IPI00216783	ubiquitin carboxyl-terminal hydrolase 2 isoform b	USP2	32	2.3	—	Q, A	P°
19	IPI00219219	Galectin-1	LGALS1	29	8.9	2.8	Q, A	P^N^
20	IPI00009636	Membrane-spanning 4-domains subfamily A member 7	MS4A7	28	7.1	—	Q, A	N
21	IPI00375617	Isoform 2 of Abhydrolase domain-containing protein 12B	ABHD12B	26	2.1	—	Q, A	P°
22	IPI00012048	Isoform 1 of Nucleoside diphosphate kinase A	NME1-NME2	24	11.2	2.73	Q, A	N
23	IPI00005685	Paraneoplastic antigen Ma1	PNMA1	22	3.7	—	Q, A	P^N^
24	IPI00022774	Transitional endoplasmic reticulum ATPase	VCP	22	3	—	Q, A	P^N^
25	IPI00470859	Putative uncharacterized protein DKFZp686C04126	MAN1B1	22	0.8	—	Q, A	P°
26	IPI00888712	actin, beta-like 3	POTEE	20	2.6	—	Q, A	P^N^
27	IPI00000816	14-3-3 protein epsilon	YWHAE	116	14.5	0.01	Q	P^N^
28	IPI00385250	Protease serine 4 isoform B	PRSS3	75	9.6	0.01	Q	P^N^
29	IPI00013508	Alpha-actinin-1	ACTN1	61	1.3	0.01	Q	P^N^
30	IPI00451401	Triosephosphate isomerase	TPI1	17	7.4	0.01	Q	P^N^
31	IPI00220642	14-3-3 protein gamma	YWHAG	58	15	0.01	Q	P^N^
32	IPI00465028	TPI1 triosephosphate isomerase isoform 2	TPI1P1	56	18.9	0.01	Q	P^N^
33	IPI00479722	Proteasome activator complex subunit 1	PSME1	56	4.4	0.01	Q	P^N^
34	IPI00453476	29 kDa protein	Null	37	15.7	0.01	Q	Null
35	IPI00021304	Keratin, type II cytoskeletal 2 epidermal	KRT2	33	4.5	0.01	Q	P^N^
36	IPI00008527	60S acidic ribosomal protein P1	RPLP1	32	14	0.01	Q	P^N^
37	IPI00021428	Actin, alpha skeletal muscle	ACTA1	26	15.6	0.01	Q	P^N^
38	IPI00479743	Isoform 1 of POTE ankyrin domain family member E	POTEKP	26	5.8	0.01	Q	P^N^
39	IPI00009791	Isoform WB of Plasma membrane calcium-transporting ATPase 2	ATP2B2	25	0.7	0.01	Q	P^N^
40	IPI00014845	Isoform 1 of Dynein heavy chain 8, axonemal	DNAH8	25	0.9	0.01	Q	P^N^
41	IPI00886949	LOC100129520, similar to hCG2044193	Null	25	4.6	0.01	Q	Null
42	IPI00002850	Hepatic leukemia factor	HLF	24	4.1	0.01	Q	P^N^
43	IPI00550020	Parathymosin	PTMS	24	10.8	0.01	Q	P^N^
44	IPI00064885	Zinc finger protein 3 homolog	ZFP3	23	1.8	0.01	Q	P^N^
45	IPI00925740	ECT2 Protein	ECT2	20	47.8	0.01	Q	P^N^
46	IPI00002349	Nuclear fragile X mental retardation-interacting protein 2	NUFIP2	19	2.6	0.01	Q	P^N^
47	IPI00218667	Stathmin-2	STMN2	19	5	0.01	Q	P^N^
48	IPI00018146	14-3-3 protein theta	YWHAQ	18	9.8	0.01	Q	P^N^
49	IPI00156689	Synaptic vesicle membrane protein VAT-1 homolog	VAT1	18	6.4	0.01	Q	P^N^
50	IPI00457114	Isoform 1 of IQ motif and SEC7 domain-containing protein 1	IQSEC1	18	3.7	0.01	Q	P^N^
51	IPI00797750	11 kDa protein	Null	17	16.7	0.01	Q	Null
52	IPI00938247	LOC100287408,hypothetical protein XP_002344194	Null	17	8.8	0.01	Q	Null
53	IPI00966637	Putative uncharacterized protein SDAD1	SDAD1	17	23.9	0.01	Q	P^N^
54	IPI00937642	LOC100290701 hypothetical protein XP_002347764	Null	15	5.6	0.01	Q	Null
55	IPI00304925	Heat shock 70 kDa protein 1A/1B	HSPA1A; HSPA1B	85	9.2	100	A	P^N^
56	IPI00830052	62 kDa protein	Null	85	10.5	100	A	Null
57	IPI00015614	Isoform A of Trypsin-3	PRSS3	78	6.6	100	A	P°
58	IPI00337495	Isoform 2 of Procollagen-lysine,2-oxoglutarate 5-dioxygenase 2	PLOD2	73	1.8	100	A	Y^N^
59	IPI00739539	POTE ankyrin domain family member F	POTEKP	63	3.6	100	A	P^N^
60	IPI00000230	tropomyosin alpha-1 chain isoform 2	TPM1	59	7.7	100	A	P°
61	IPI00013991	Isoform 1 of Tropomyosin beta chain	TPM2	59	8.8	100	A	P°
62	IPI00026260	Isoform 1 of Nucleoside diphosphate kinase B	NME1-NME2	59	25	100	A	N
63	IPI00879437	Protein disulfide-isomerase A1	P4HB	54	16.5	100	A	P°
64	IPI00937995	Actin-like protein (Fragment)	ACTB	50	19.4	100	A	P°
65	IPI00024320	Putative RNA-binding protein 3	RBM3	40	20.4	100	A	P°
66	IPI00171611	Histone H3.2	HIST2H3A	40	11.8	100	A	P^N^
67	IPI00798360	SARNP 18 kDa protein	SARNP	40	4.3	100	A	P^N^
68	IPI00878173	cDNA FLJ39583 fis, clone SKMUS2004897, highly similar to ACTIN, ALPHA SKELETAL MUSCLE	ACTA1	38	15.2	100	A	P°
69	IPI00045396	Calumenin isoform 4	CALU	34	2.2	100	A	Y^N^
70	IPI00016768	L-lactate dehydrogenase A-like 6B	LDHAL6B	31	4.5	100	A	P^N^
71	IPI00784327	Isoform 1 of 1-phosphatidylinositol-4,5-bisphosphate phosphodiesterase beta-2	PLCB2	30	1.9	100	A	P^N^
72	IPI00000874	Peroxiredoxin-1	PRDX1	29	10.1	100	A	P^N^
73	IPI00216817	HEAT repeat containing 7B1	HEATR7B1	29	1.3	100	A	P^N^
74	IPI00293975	16 kDa protein	Null	29	6.2	100	A	Null
75	IPI00298622	Intestinal-type alkaline phosphatase	ALPI	29	2.1	100	A	Y^N^
76	IPI00945706	BOC Protein	BOC	29	14.3	100	A	P^N^
77	IPI00329801	Annexin A5	ANXA5	26	2.8	100	A	P°
78	IPI00014424	Elongation factor 1-alpha 2	EEF1A2	25	3.9	100	A	P^N^
79	IPI00030282	Isoform 1 of Filensin	BFSP1	25	3.9	100	A	P°
80	IPI00910689	Polyserase-2	PRSS36	25	3.2	100	A	Y^N^
81	IPI00023673	Galectin-3-binding protein	LGALS3BP	24	3.8	100	A	Y^N^
82	IPI00414717	Isoform 2 of Golgi apparatus protein 1	GLG1	24	2.1	100	A	Y^N^
83	IPI00007249	Ectonucleotide pyrophosphatase/phosphodiesterase family member 4	ENPP4	23	5.7	100	A	Y^N^
84	IPI00008692	Isoform 1 of Keratin, type I cuticular Ha6	KRT36	23	4.9	100	A	P^N^
85	IPI00290077	Keratin, type I cytoskeletal 15	KRT15	23	4.4	100	A	P^N^
86	IPI00384497	Protein-tyrosine phosphatase-like member B	PTPLB	23	7.1	100	A	Y^N^
87	IPI00908888	cDNA FLJ57836, highly similar to Myb-binding protein 1A	MYBBP1A	23	2.2	100	A	P^N^
88	IPI00024658	OTU domain-containing protein 7B	OTUD7B	22	2.5	100	A	P°
89	IPI00384972	Isoform 1 of MLL1/MLL complex subunit KIAA1267	KIAA1267	22	1.5	100	A	P^N^
90	IPI00394814	Serine protease 55	PRSS55	22	4.8	100	A	Y^N^
91	IPI00216704	Isoform 2 of Spectrin beta chain, erythrocyte	SPTB I	21	1.7	100	A	P^N^
92	IPI00455675	Centrosomal protein of 192 kDa	CEP192	20	1.1	100	A	P^N^
93	IPI00552749	DNAH8 478 kDa protein	DNAH8	20	1.7	100	A	P^N^
94	IPI00748715	SEPT9 protein (Fragment)	Null	19	3.5	100	A	Null
95	IPI00020035	Protein NipSnap homolog 3B	NIPSNAP3B	18	12.6	100	A	P°
96	IPI00217899	E3 ubiquitin-protein ligase RNF168	RNF168	18	1.9	100	A	P^N^
97	IPI00010289	D(1A) dopamine receptor	DRD1	17	4	100	A	Y^N^
98	IPI00478586	Isoform 2 of Vacuolar protein sorting-associated protein 13A	VPS13A	17	1.9	100	A	P^N^
99	IPI00929137	Conserved hypothetical protein	Null	17	9.9	100	A	Null
100	IPI00011500	Isoform 1 of Testicular acid phosphatase	ACPT	16	2.8	100	A	Y^N^
101	IPI00299507	Condensin complex subunit 2	NCAPH	16	0.9	100	A	P^N^
102	IPI00009101	Isoform 2 of Helicase SRCAP	SRCAP	15	1	100	A	P^N^
103	IPI00442299	Isoform 1 of Neurexin-1-alpha	NRXN1	15	3.3	100	A	Y^N^
104	IPI00947233	PCOLCE2 Protein	PCOLCE2	14	42.6	100	A	P^N^
105	IPI00294052	Single-stranded DNA-binding protein 2	SSBP2	13	3.6	100	A	P^N^

^a^: Glycoproteins with an emPAI ratio of >2.0 or <0.5 are shown. “A/Q” represents the ratio of the emPAI value of a protein in the activated LX-2 cells *vs.* that in the quiescent LX-2 cells. The “A/Q” value of glycoproteins identified specifically in quiescent LX-2 was assigned as 0.01, while that of glycoproteins identified specifically in the activated LX-2 was assigned as 100; ^b^: Data in column “Q or A” represent the CBG identified in the quiescent LX-2 (Q) or the activated LX-2 (A); ^c^: “Y^N^” represents the CBGs annotated as “N-linked glycosylated” in Swiss-Prot; “Y°” represents the CBGs annotated as “O-linked glycosylated” in Swiss-Prot; “P^N^” represents potential N-linked glycoproteins predicted by the software NetNGlyc 1.0 Server; “P°” represents potential O-linked glycoproteins predicted by the software NetOGlyc 4.0 Server; “N” represents proteins with no typical glycosylation site.

### 2.3. GO Classification of the Identified CBGs Using Blast2GO^®^

**Figure 2:**
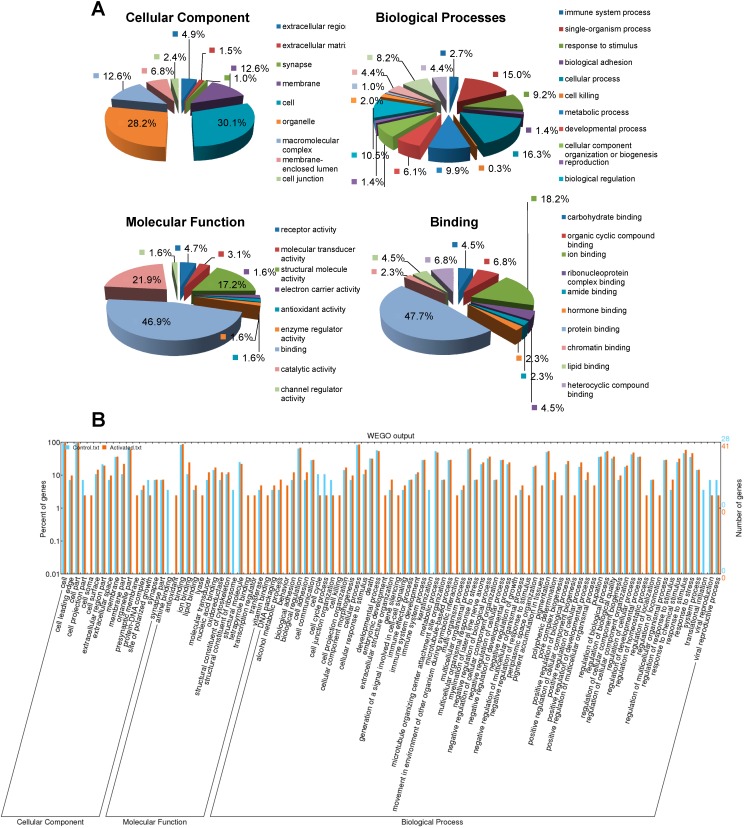
The GO annotations of the identified CBGs in LX-2 cells. (**A**) Total CBGs identified in LX-2 cells were analyzed for functional enrichment according to the three grouping classifications of cellular component, biological process, and molecular function using the software Blast2GO^®^; (**B**) Gene ontology (GO) classification and comparison of enrichment of functional groups between the quiescent and the activated LX-2 cells was performed using WEGO software.

To investigate the major biological functions of CBGs in LX-2 cells, the commercially available software program Blast2GO was used to analyze the proteins that were identified in LX-2 cells for functional enrichment according to three grouping classifications: cellular components, biological processes, and molecular functions (Figure 2A). Of 105 identified CBGs, 98 had gene ontology (GO) annotations available. In terms of cellular components, 62 proteins (30.1%) were grouped as cell proteins, while 58 proteins (28.2%) were grouped as organelle proteins. Moreover, other protein groupings were found, with groups including: cell membrane (26, 12.6%), macromolecular complex (26, 12.6%), membrane-enclosed lumen (14, 6.8%), and extracellular region (10, 4.9%). In terms of biological processes, about 48 proteins (16.3%), 44 proteins (15.0%), 31 proteins (10.5%), 29 proteins (9.9%), and 27 proteins (9.2%) were included in the groups of cellular process, single-organism process, biological regulation, metabolic process, and response to stimulus, respectively. In terms of molecular function, proteins with binding ability formed the largest group (30, 43.9%) and other smaller groups identified included catalytic activity (14, 21.9%), structural molecule activity (11, 17.2%), and receptor activity (3, 4.7%). Detailed analysis of the binding ability group revealed that proteins with the ability to bind to proteins (21, 47.7%) and ions (8, 18.2%) comprised the most abundant subgroups. To determine whether there were any differences in the three aspects of GO annotations between activated and quiescent LX-2 cells, GO enrichment analysis was performed. Greater enrichment was found in the response to stimulus, detection of stimulus, activation of immune response, and gene silencing groups, while enrichment of the translation initiation and cell cycle process groups was much lesser in activated LX-2 cells than in the quiescent control cells (Figure 2B).

In our previous study, we found large differences in the distribution of glycoproteins bound among ConA and other lectins in LX-2 cells. The glycoproteins recognized by *Aleuria aurantia* lectin (AAL), *Erythrina cristagalli* lectin (ECA), and phytohemagglutinin (PHA-E) were mainly located on the cytoplasmic membrane and the perinuclear cytoplasm (*i.e.*, the endoplasmic reticulum [ER] and Golgi complex), while ConA showed positive binding to the glycoproteins located in the whole cytoplasm and/or cytoplasmic membrane [18]. In the present study, 62 proteins (30.1%) were grouped as intracellular proteins and 58 (28.2%) as organelle proteins. Most of the proteins in the organelle proteins group were also included in the intracellular proteins group. It is known that the common protein-bound glycan adduct, N-linked core oligosaccharide, is composed of two N-acetylglucosamine, nine mannose, and three glucose residues, and this oligosaccharide molecule is synthesized and processed in the ER [37]. Glycans are subjected to extensive modifications as glycoproteins mature and move through the ER via the Golgi complex to their final destinations inside and outside the cell [38]. Con A can specifically recognize the trimannoside core of the N-glycan [39], so N-glycosylated proteins that are still undergoing processing and sorting in the ER and Golgi can also be recognized by ConA, in addition to the mature membrane and secreted glycoproteins. For some organelle proteins, such as Golgi glycoprotein 1 (GLG1) (Table 1) and lysosomal enzymes, their synthesis and positioning also depend on the glycosylation associated with ER-Golgi trafficking [40,41]. In addition, O-glycosylations such as O-GlcNAc, which contribute to nutrient sensing and regulation of insulin signaling, cell cycle, calcium signaling pathway, and cellular stress response [42,43,44], may contain multiple mannose residues that can also be recognized by ConA, since CBGs were distributed not only in the perinuclear cytoplasm of the LX-2 cells, but also in the whole cytoplasm and cell membrane.

### 2.4. KEGG Pathway Analysis and Functional Protein Association Networks

**Figure 3:**
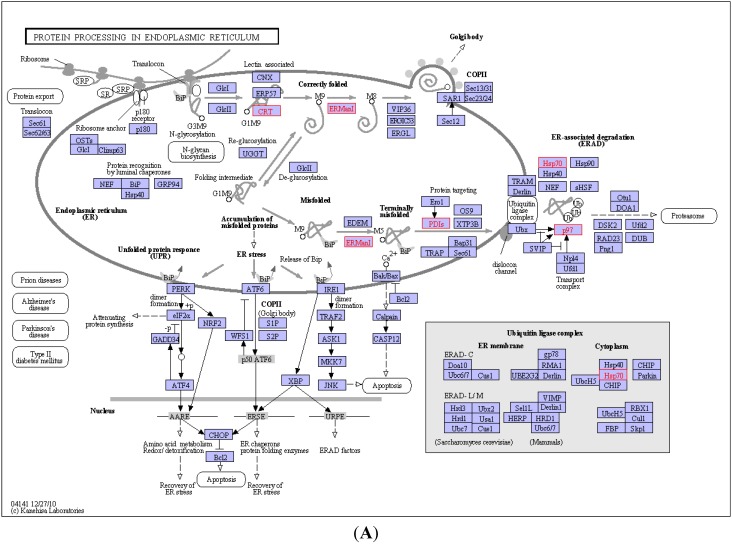

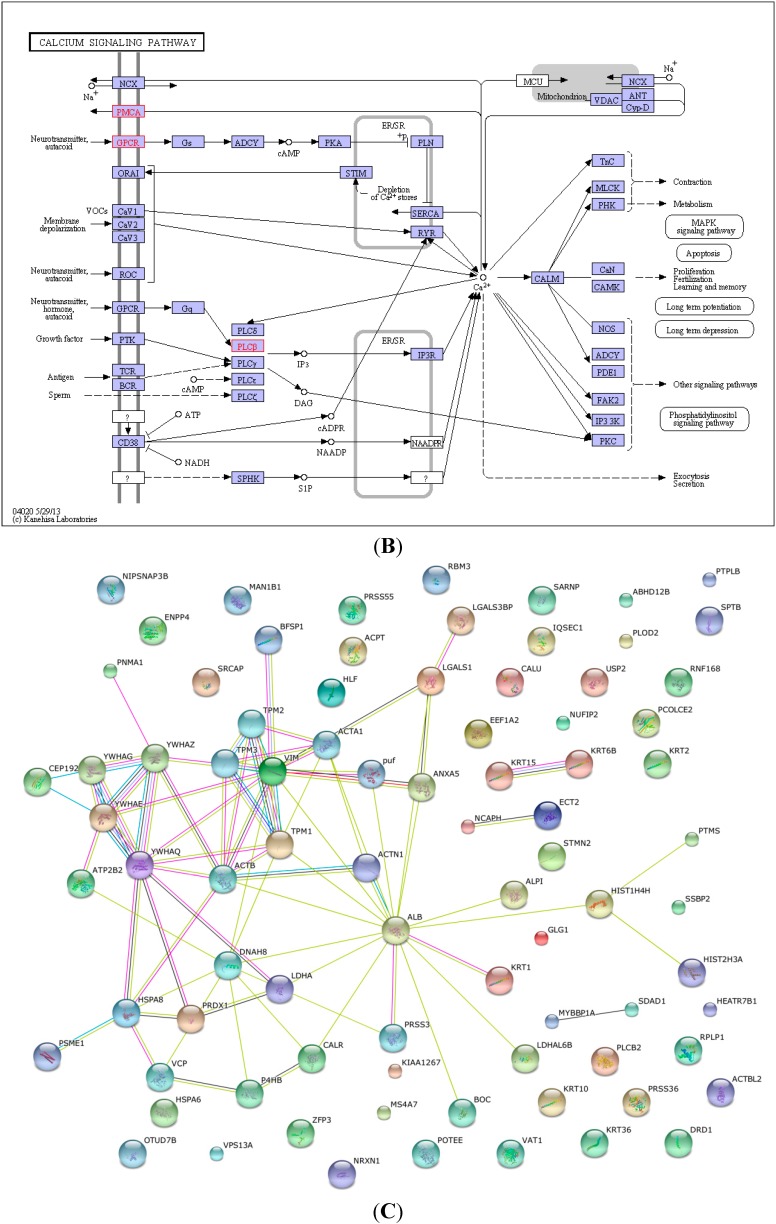

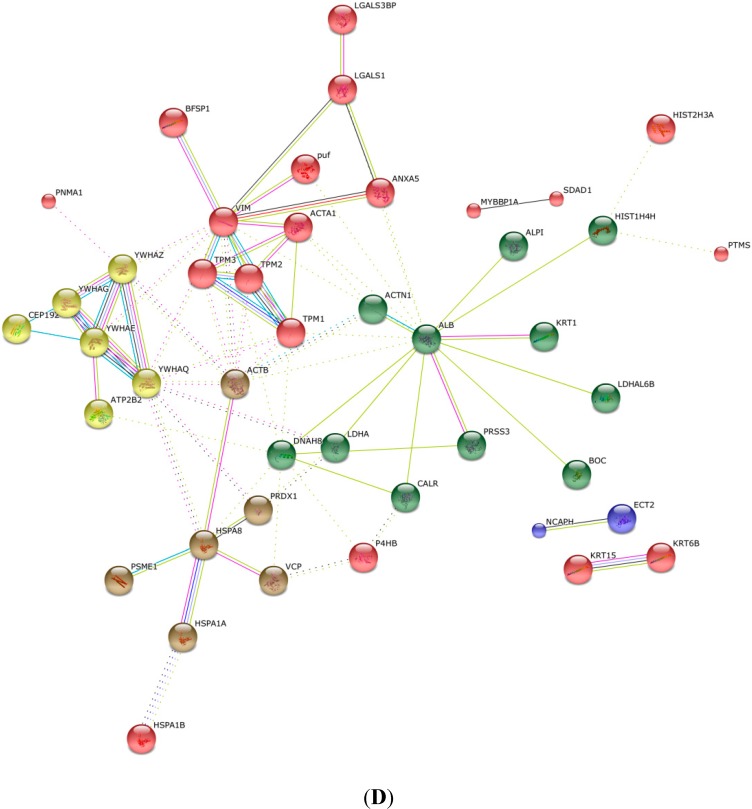
KEGG pathway analysis and functional protein association networks. (**A**,**B**) show the CBGs mapped to the KEGG pathways of protein processing in the ER and calcium signaling. The CBGs involved in these networks are labeled with a red frame; (**C**,**D**) display the potential interactions among total CBGs and show the CBGs that were determined to show significant correlations by STRING analysis.

In total, 91 of 105 identified CBGs had been annotated in DAVID Bioinformatics Resources version 6.7. These glycoproteins were mapped to 6 KEGG pathways with the thresholds of count of >2 and *p* value of <0.05 compared with the background signal of the human genome; the identified KEGG pathways included protein processing in the ER, calcium signaling pathway, cell cycle, glycolysis/gluconeogenesis, and others (Figure 3A,B, Table S1). Proteins involved in protein processing in the ER (e.g., CALR, protein disulfide-isomerase A1, and heat shock 70-kDa protein 1A/1B) and calcium signaling pathway (e.g., D[1A] dopamine receptor [DRD1] and 1-phosphatidylinositol-4,5-bisphosphate phosphodiesterase β-2) were specifically identified or upregulated in the activated LX-2 cells. In contrast, 14-3-3 protein family members (e.g., 14-3-3 ζ/δ, 14-3-3 ε, and 14-3-3 γ) involved in the cell cycle and the neurotrophin signaling pathway were specifically identified in the quiescent LX-2 cells.

A total of 90 matched CBGs were queried against the STRING *Homo sapiens* database to determine their functional relevance (Figure 3C). Through K-mean clustering analysis, associations among TPM1, TPM2, ACTB, DNAH8, and ACTA1; LGALS1, ANXA5, and LGALS3BP; P4HB and CALR; and HSPA1B and HSPA1A were specifically identified in the activated LX-2 cells, whereas associations among YWHAZ, YWHAE, YWHAG, and YWHAQ were specifically identified in the quiescent LX-2 cells (Figure 3D).

The aims of this study were not only to find novel CBGs that differentially expressed in the activated HSCs, but also to speculate the possible pathway networks associated with fibrogenesis in HSCs. Proteins involved in protein processing in ER and calcium signaling pathway were higher expressed in the activated HSCs (Figure 3A,B, and Table S1), which partially demonstrated that these pathways were activated in HSCs when stimulated by TGF-β1. Interestingly, the expression levels of galectin-1 (LGALS1) and galectin-3-binding protein (LGALS3BP) were upregulated or specifically identified in the activated LX-2 cells (Table 1). The functions of galectins have been reported to be involved in physiological and pathological processes of the liver [45,46]. A previous proteomics analysis of rat HSC proteins revealed that the production and secretion of LGALS1 is greatly increased in activated HSCs compared to that in quiescent HSCs [45]. LGALS3 expression was found to be induced in regenerative nodules of liver cirrhosis tissues and in hepatocellular carcinomas [47]. A further study demonstrated that both LGALS1 and LGALS3 activate mitogen-activated protein kinase (MAPK) pathways, presumably by forming cross-links with target molecules through their β-galactoside-containing glycoconjugates, leading to the proliferation of HSCs [48]. In addition, an increased concentration of cytoplasm Ca^2+^ can also activate the Ca^2+^/calmodulin-dependent protein kinase (CaMKII)/MAPK signaling pathway [49]. Intracellular free Ca^2+^ is a crucial second messenger that plays various roles in regulating a wide range of cellular processes in different cells and tissues. To maintain a low Ca^2+^ concentration of 10–100 nM in the cytoplasm, Ca^2+^ is actively pumped from the cytoplasm to the extracellular space and ER, and sometimes into the mitochondria. Specific signals can trigger an increase in the cytoplasmic Ca^2+^ level up to 500–1000 nM by opening channels in the plasma membrane or ER [50]. DRD1 is a G protein-coupled receptor that stimulates adenylate cyclase and was found to inhibit Ca^2+^ from being pumped from the cytosol to the ER [51]. The plasma membrane Ca^2+^ ATPase (ATP2B) is a transport protein in the plasma membrane of cells that serves to remove Ca^2+^ from the cytosol [52]. 1-phosphatidylinositol-4,5-bisphosphate phosphodiesterase β-2 (PLCB2) participates in phosphatidylinositol 4,5-bisphosphate (PIP2) metabolism and indirectly promotes the release of Ca^2+^ from the ER to the cytosol [53]. According to our results, DRD1 and PLCB2 were specifically identified in the activated LX-2 cells, whereas ATP2B was specifically identified in the quiescent LX-2 cells, indicating that Ca^2+^ became more inclined to accumulation in the cytoplasm, rather than the extracellular space or the ER in the LX-2 cells activated with TGF-β1 (Figure 3B). Thus, it can be conjectured that increases in galectin expression and intracellular Ca^2+^ concentration might collectively activate the MAPK signaling pathway to realize the activation of HSC induced by TGF-β1. This view is different from the traditional view that platelet derived growth factor (PDGF) induced the activation of HSCs through MAPK signaling pathway [54] and TGF-β1 through TGFβ1/ALK1/Smad1 pathway, and might be therapeutic targets of liver fibrosis. Actually, a recent study suggested that the production of CTGF by TGF-β activated MAPK signaling in hepatic progenitor cells [55]. However, the detailed molecular mechanisms of these cytokines still need to be further studied.

### 2.5. Expression of PLCB2 in Activated LX-2 Cells and Human Liver Cirrhosis Tissue

**Figure 4:**
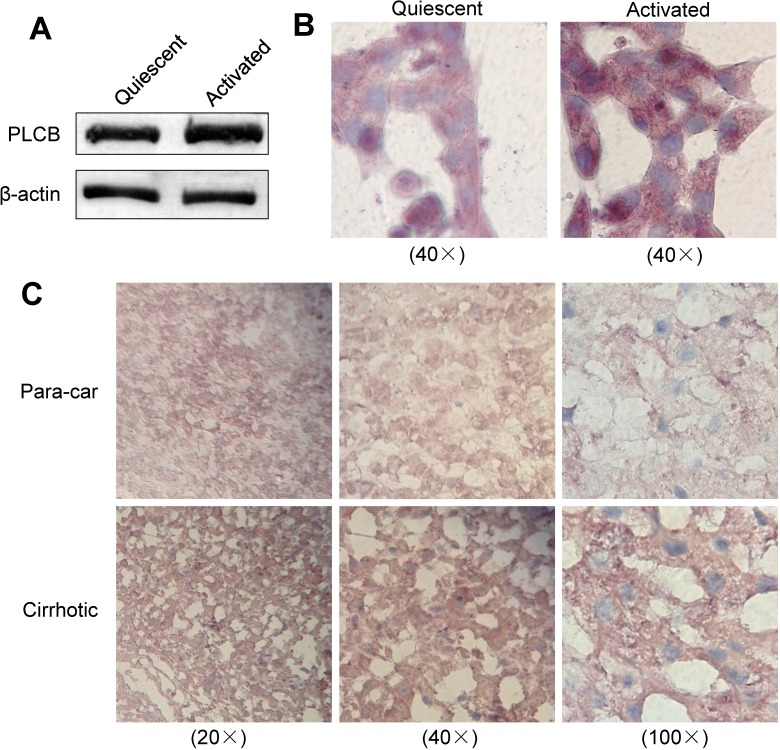
Expression of PLCB2 in the activated LX-2 cells and human liver cirrhosis tissue. (**A**) Western blots showed that the expression of PLCB2 was significantly increased in the activated, compared with quiescent LX-2 cells; (**B**) Immunohistochemistry to validate and investigate the expression and location of PLCB2 in the quiescent and the activated LX-2 cells. The images were shown on the same scale for the quiescent and activated LX-2 cells (images were obtained using a 40× objective lens); (**C**) Immunohistochemistry to investigate the expression and location of PLCB2 in liver cirrhosis tissues and the normal para-carcinoma tissues. The images were taken using 20×, 40×, and 100× objective lenses, respectively.

To further validate and investigate the expression of CBGs and their relationship to liver fibrosis and cirrhosis, the increased expression of PLCB2, which was specifically identified in the activated LX-2 cells, was measured in LX-2 cells and human liver by western blotting and immunohistochemistry, respectively. The expression of PLCB2 was found to be distinctly increased in activated, compared with quiescent LX-2 cells (Figure 4A,B) and was also increased in liver cirrhosis tissues compared with normal para-carcinoma tissues (Figure 4C). PLCB2 was found to be mainly distributed in the cytoplasm of the activated LX-2 cells (Figure 4B), as well as that of hepatocytes and sinusoidal cells in liver tissues (Figure 4C). These results were consistent with the MS analysis and provided further evidence indicating a possible vital function of PLCB2 in the development of liver fibrosis and cirrhosis.

In this study, 62 proteins (30.1%) were intracellular proteins and 26 (12.6%) were located on the cell membrane, in accordance with the result that ConA showed strong binding to the cytoplasm and/or membrane of LX-2 cells in our previous study [18]. The activation of HSCs is a complex and persistent response to chronic liver injury. In the initial stages, HSCs receive paracrine stimulation from cytokines secreted by neighboring cells such as Kupffer cells, hepatocytes, and leukocytes. In the persistent stages, autocrine cytokines (e.g., TGF-β and connective tissue growth factor) persistently stimulate HSCs so as to regulate and maintain their activation [56,57]. In this study, a greater number of CBGs were identified from the activated LX-2 cells than from the quiescent LX-2 cells. In addition, glycoproteins involved in the biological processes of response to stimulus, detection of stimulus, and activation of immune response were upregulated in the activated LX-2 cells. For example, PLCB2 was validated to be upregulated in the cytoplasm of the activated LX-2 cells, as well as in the hepatocytes and sinusoidal cells of liver cirrhosis tissues. Based upon the above findings, CBGs might play important roles in signal conduction and response to stimulus, and be associated with the formation and development of liver fibrosis and cirrhosis.

## 3. Experimental Section

### 3.1. Materials

Dulbecco’s modified Eagle’s medium (DMEM) and fetal bovine serum (FBS) were purchased from Invitrogen (Carlsbad, CA, USA). TGF-β1 was purchased from R&D systems (Minneapolis, MN, USA). Con A was purchased from Vector Laboratories (Burlingame, CA, USA). DTT and iodoacetamide were purchased from GE Healthcare (Little Chalfont, Buckinghamshire, UK). Mouse anti-PLCB2 monoclonal antibody, urea, ammonia bicarbonate (NH_4_HCO_3_), sodium periodate (NaIO_4_), HPLC-grade acetonitrile (ACN) and trifluoroacetic acid (TFA), protease inhibitor cocktail, and Bradford assay reagent were purchased from Sigma-Aldrich (St. Louis, MO, USA). Trypsin was purchased from Promega (Madison, WI, USA). Peptide-N-glycosidase F (PNGase F) was purchased from New England Biolabs (Ipswich, MA, USA). Bovine serum albumin (BSA) was purchased from Merck (Darmstadt, Germany). T-PER^®^ reagent was purchased from Pierce Biotechnology (Rockford, IL, USA). Other chemical reagents were obtained from commercial suppliers and used without further purification. All of the solutions were prepared with ultra-pure water obtained from a Milli-Q^®^ 50 SP Reagent Water System (Millipore, Bradford, MA, USA).

Amicon Ultra-0.5 10-kDa devices were obtained from Millipore. C18 SepPak^®^ columns were from Waters (Milford, CT, USA). The ZHWY- 2101C Oscillator was purchased from ZhiCheng Corporation, China. The SpeedVac^TM^ vacuum concentrator was obtained from Thermo Scientific (Waltham, MA, USA). Nanospray CHIP-LC-MS/MS 6530 mass spectrometers equipped with HP 1200 solvent delivery systems were purchased from Agilent (Santa Clara, CA, USA).

### 3.2. Cell Culture and Stimulation

The LX-2 cells were a gift from Dr. Friedman of the Mount Sinai School of Medicine, New York, NY, USA. Cells were cultured and stimulated as described previously [18,58]. About 2 × 10^5^ LX-2 cells were seeded and rested in high-glucose DMEM supplemented with 10% (v/v) FBS for 24 h. After 24 h of starvation, cells were stimulated by adding 2 ng/mL TGF-β1 for 24 h. The control (quiescent) groups were not treated with TGF-β1.

### 3.3. Non-Denaturing Extraction of Total Proteins

Total protein was extracted from LX-2 cells using non-denaturing T-PER reagent as described previously [18]. The adherent cells were washed twice with ice-cold 1× PBS (0.01 M phosphate buffer containing 0.15 M NaCl, pH 7.4) to remove culture medium. The cells were incubated for 15 min with T-PER reagent mixed with protease inhibitors (10 μL/mL T-PER reagent) and then homogenization was achieved by triturating cells with a pipette. Following centrifugation at 10,000× *g* for 10 min, the supernatants were either immediately collected for use or stored at −80 °C. The protein concentration in the supernatants was determined by Bradford assay.

### 3.4. Isolation of Glycoproteins from LX-2 Cells Using ConA-Magnetic Particle Conjugates (CMPCs)

A subset of glycoproteins binding to ConA were selectively isolated by CMPCs as described previously [59,60]. Briefly, 2 mg of total proteins from LX-2 cells were diluted into 600 µL of binding buffer (0.1 M Tris-HCl, 150 mM NaCl, 1 mM CaCl_2_, 1 mM MgCl_2,_ and 1 mM MnCl_2_, pH 7.4) supplemented with 1% (v/v) proteinase inhibitor. The homemade ConA-magnetic particle conjugates (CMPCs) were rinsed three times with binding buffer and then incubated with proteins at room temperature for 3 h under gentle shaking. The unbound proteins were removed by washing three times with the rinsing buffer (binding buffer with the addition of 0.1% (v/v) Tween 20) thoroughly. Then, the glycoproteins bound to CMPCs were eluted with 300 µL of the elution buffer (binding buffer supplemented with 0.5 M methyl-α-d-mannose, 0.2 M methyl-α-d-glucose, and 1% (v/v) proteinase inhibitor) at room temperature for 1 h under gentle shaking, and the supernatants were collected.

### 3.5. Identification of Peptides by LC-MS/MS

The glycoproteins isolated by CMPCs were concentrated and desalted by a size-exclusion spin filter (Amicon Ultra-0.5 10 K device), with a molecular mass cutoff of 10 kDa. The obtained glycoproteins were denatured in 8 M urea, then deoxidated with 10 mM DTT and carboxyamidomethylated with 20 mM iodoacetamide. Subsequently, trypsin was added at a 1:100 (w/w) ratio of enzyme to protein and the samples were incubated overnight at 37 °C. Glacial acetic acid (5 µL, pH 2.0) was added to stop the reaction, samples were centrifuged, and the supernatants were collected and lyophilized. Then, the N-linked glycans of the glycopeptides were released by incubation with PNGase F overnight at 37 °C. The peptides were desalted using C18 SepPak columns and lyophilized. Finally, the peptides were resolubilized in 0.1% (v/v) formic acid and analyzed using nanospray CHIP-LC-MS/MS 6530 mass spectrometers equipped with HP 1200 solvent delivery systems.

### 3.6. Label-Free Relative Quantification of Identified Glycoproteins by emPAI

Label-free quantification methods are popular because of their convenience and accuracy. Mascot search engine software (Matrix Science, Boston, MA, USA) includes a semi-quantification method, emPAI, which is a convenient tool for relative quantification [61]. To compare the activated *versus* quiescent LX-2 glycoproteomes, we calculated normalized emPAI values for each protein (see Formula (1)), and estimated the fold-change in the activated *versus* quiescent LX-2 cells. When a protein was not identified in the quiescent LX-2 cells, we arbitrarily assigned the fold-change as 100 to avoid dividing by zero.

normalized emPAI of a protein = emPAI of that protein/sum of all emPAIs in a sample × 100
(1)

### 3.7. Data Mining and Bioinformatics

Ontology analysis was performed according to the standard procedure of Blast2GO [62] to gain insights into functional groupings within the set of identified glycoproteins in three aspects of GO. The differences in the GO terms of glycoproteins between the activated and quiescent LX-2 cells were also discovered by analysis using WEGO [63]. Biological pathways that were enriched in the identified glycoproteins originated from the KEGG human pathway database [64]. To validate the results of Blast2GO and KEGG, an independent functional enrichment analysis was performed using DAVID (Database for Annotation, Visualization and Integrated Discovery) [65]. Finally, to determine the functional relevance of the identified glycoproteins, we performed an analysis of protein association networks using STRING 9.05 [66].

### 3.8. SDS-PAGE and Western Blotting

For SDS-PAGE, protein samples were mixed with 5× loading buffer and boiled for 4 min at 100 °C, and then separated on a 10% polyacrylamide resolving gel and a 3% stacking gel. Molecular mass standards (Thermo Scientific) were run for each gel. Gels were then stained directly with alkaline silver. For western blotting, 30 μg of proteins were separated by 10% SDS-PAGE and then transferred to PVDF membranes (Millipore). After blocking for 2 h at room temperature with 5% (w/v) skimmed milk powder in TBS (100 mM Tris-HCl, 150 mM NaCl, pH 7.6), the membrane was incubated overnight at 4 °C with mouse monoclonal antibodies against PLCB2 in antibody dilution buffer (5% (w/v) skimmed milk in TBST (0.05% (v/v) Tween 20 in TBS, pH 7.6)). The membranes were washed three times with TBST, and then incubated with alkaline phosphatase-conjugated secondary antibodies (1:1000 (v/v) dilution in antibody dilution buffer) for 1 h at room temperature. The membranes were washed three times with TBST and detected using a 5-bromo-4-chloro-3-indolyl phosphate/nitroblue tetrazolium salt substrate kit. β-Actin was used as an internal control.

### 3.9. Immunohistochemistry

Immunohistochemistry was performed as described previously [67]. Briefly, 2 × 10^5^ LX-2 cells were seeded in 6-well culture plates containing sterile coverslips. The adherent cells were fixed with 4% (w/v) paraformaldehyde and permeabilized in ice-cold 1× PBS supplemented with 1% (v/v) Triton^TM^ X-100 at 4 °C for 10 min and rinsed twice in 1× PBS. The permeabilized cells were then treated with hydrogen peroxide (H_2_O_2_) (3% (v/v) in methanol) for 20 min to block endogenous peroxidase. Prior to incubation with anti-PLCB2, cells were blocked with blocking solution (10% (w/v) BSA in 1× PBS) for 1 h at room temperature. Then, cells were incubated with PLCB2 antibody diluted at 1:1000 in blocking solution overnight at 4 °C. Then, cells were incubated with alkaline phosphatase-conjugated secondary antibodies in blocking solution for 1 h at room temperature, and horseradish peroxidase colorimetric development solution was applied according to the manufacturer’s instructions. Finally, the cells were further stained with hematoxylin and histological images were obtained.

## 4. Conclusions

The profile of CBGs in human HSCs (LX-2 cell line) activated with TGF-β1 was compared with that of quiescent HSCs by employing a glycoproteomics approach using a CMPCs coupled MS technique. Novel CBGs and the signaling pathways/networks in which they were involved were discovered and partially validated to be connected with the activation of HSCs. The results of this study may provide useful information to aid future investigation to identify new molecular mechanisms for HSC activation and antifibrotic therapeutic targets.

## Supplementary Materials

Supplementary materials can be accessed at: http://www.mdpi.com/1420-3049/19/12/19845/s1.
