# Supplementary material for: Profiling of Concanavalin A-Binding Glycoproteins in Human Hepatic Stellate Cells Activated with Transforming Growth Factor-β1
Source: Molecules. 2014 Nov 28;19(12):19845–67. doi: 10.3390/molecules191219845 (PMC6270946; doi:10.3390/molecules191219845)
Supplement: Supplementary file 1 [file molecules-19-19845-s001.pdf]

Supplementary Materials

Table S1. Glycoproteins annotated in DAVID Bioinformatics Resources were mapped to six KEGG pathways.

Category	Term	Count	*P* Value	Genes	Fold Enrichment
KEGG_PATHWAY	hsa04612:Antigen processing and presentation	5	0.0017	HSPA8, 62 kDa protein, CALR, HSPA1A/1B, PSME1	9.28
KEGG_PATHWAY	hsa04020: calcium signaling pathway	3	0.0063	DRD1, PLCB2, ATP2B	6.34
KEGG_PATHWAY	hsa04722:Neurotrophin signaling pathway	4	0.042	YWHAE, YWHAG, YWHAZ, YWHAQ	4.97
KEGG_PATHWAY	hsa04110:Cell cycle	4	0.043	YWHAE, YWHAG, YWHAZ, YWHAQ	4.93
KEGG_PATHWAY	hsa00010:Glycolysis/Gluconeogenesis	3	0.049	TPI1P1, LDHA, LDHAL6B	7.70
KEGG_PATHWAY	hsa04520:Adherens junction	2	0.050	ACTN1, ACTB	6.00
